# Circulating leptin levels are associated with adiposity in survivors of childhood brain tumors

**DOI:** 10.1038/s41598-020-61520-2

**Published:** 2020-03-13

**Authors:** E. Danielle Sims, William J. Jennings, Brianna Empringham, Adam Fleming, Carol Portwine, Donna L. Johnston, Shayna M. Zelcer, Shahrad Rod Rassekh, Sarah Burrow, Lehana Thabane, M. Constantine Samaan

**Affiliations:** 1 Department of Pediatrics, McMaster University, Hamilton, Ontario Canada; 2 Division of Pediatric Endocrinology, McMaster Children’s Hospital, Hamilton, Ontario Canada; 3 Division of Pediatric Hematology/Oncology, McMaster Children’s Hospital, Hamilton, Ontario Canada; 4 Division of Pediatric Hematology/Oncology, Children’s Hospital of Eastern Ontario, Ottawa, Ontario Canada; 5 Pediatric Hematology Oncology, Children’s Hospital, London Health Sciences Center, London, Ontario Canada; 6 Division of Pediatric Hematology/Oncology/BMT, Department of Pediatrics, British Columbia Children’s Hospital, Vancouver, BC Canada; 7 Division of Orthopedic Surgery, Department of Surgery, McMaster University Medical Centre, Hamilton, Ontario Canada; 8 Department of Health Research Methods, Evidence and Impact, McMaster University, Hamilton, Ontario Canada; 9 Department of Anesthesia, McMaster University, Hamilton, Ontario Canada; 10 Centre for Evaluation of Medicines, St. Joseph’s Health Care, Hamilton, Ontario Canada; 11 Biostatistics Unit, St Joseph’s Healthcare-Hamilton, Hamilton, Ontario Canada

**Keywords:** Molecular biology, Predictive markers, Endocrine system and metabolic diseases

## Abstract

Survivors of Childhood Brain Tumors (SCBT) are at a higher risk of developing cardiovascular disease and type 2 diabetes compared to the general population. Adiposity is an important risk factor for the development of these outcomes, and identifying biomarkers of adiposity may help the stratification of survivors based on their cardiovascular risk or allow for early screening and interventions to improve cardiometabolic outcomes. Leptin is an adipokine that positively correlates with the adipose mass in the general population and is a predictor of adverse cardiometabolic outcomes, yet its association with adiposity in SCBT has not been studied. The aim of this study was to determine if leptin levels are associated with the adipose mass in SCBT, and to define its predictors. This cross-sectional study included 74 SCBT (n = 32 females) with 126 non-cancer controls (n = 59 females). Total adiposity was measured using Bioelectrical Impendence Analysis (BIA) and central adiposity was measured using waist-to-hip ratio (WHR) and waist-to-height ratio (WHtR). We used multivariable linear regression analysis to determine if leptin predicts adiposity in SCBT and adjusted for age, sex, puberty, and cancer status. Leptin correlated strongly with total (p < 0.001) and central (WHR p = 0.001; WHtR p < 0.001) adiposity in SCBT and non-cancer controls. In conclusion, leptin is a potential biomarker for adiposity in SCBT, and further investigation is needed to clarify if leptin is a predictor of future cardiometabolic risk in SCBT.

## Introduction

Cardiovascular disease is a leading cause of morbidity and mortality, accounting for approximately 17.6 million deaths per annum globally^1,2^. While cardiovascular diseases are a significant burden on healthcare systems in the general population^1–4^, specific populations seem to have a higher propensity for these diseases than others, and one such cohort includes the survivors of childhood cancer. Cardiovascular diseases are one of the leading causes of non-cancer related mortality in this population, and accounts for 20% of mortality rates within 15 years of cancer therapy^5–8^.

Within the cancer survivorship subgroups, the Survivors of Childhood Brain Tumors (SCBT) have a significant risk of premature cardiovascular diseases and one of their major risk factors, type 2 diabetes mellitus^6,9,10^. SCBT have a 29-fold higher risk for stroke and a two-fold higher risk of type 2 diabetes compared to non-cancer controls^9,11^. Cardiometabolic disorders are emerging as an important determinant of longevity and quality of life in SCBT^8^, and there is a critical necessity to identify the risk factors and biomarkers of cardiometabolic risk to personalize preventative and therapeutic strategies to improve outcomes in this population.

The presence of excess adiposity is a major risk factor for cardiovascular diseases and type 2 diabetes mellitus in the general population^12,13^. Importantly, SCBT have an important phenotypic difference when compared to non-cancer controls with excess total and central adiposity in the presence of similar Body Mass Index (BMI)^14–18^. This is critically important, as higher adiposity during childhood carries over to adulthood and is associated with adverse cardiometabolic outcomes in the general population, but the path to excess adiposity is unknown in SCBT group^19–22^.

Leptin is an adipokine that serves as a biomarker of the fat mass in the general population^23–26^, and hyperleptinemia is a predictor of several cardiometabolic outcomes^27–31^ including diabetes^27,28^, glucose intolerance^29^, insulin resistance^29^, coronary events^30^, hypertension^31^, and features of the metabolic syndrome^29^. However, the relationship between leptin levels, adiposity, and cardiometabolic outcomes in SCBT is not known. In this paper, we tested the hypothesis that leptin is associated with adiposity in SCBT in a similar way to this association in non-cancer controls. We also set out to define the potential predictors of leptin levels in SCBT.

## Results

### Demographics

The population characteristics are reported in Table 1. The SCBT group was recruited at 6 ± 4.2 years post completion of cancer therapy, and included 74 SCBT (n = 32 female, 43.20%) and 126 non-cancer controls (n = 59 female, 46.80%). The groups had similar age distribution (SCBT: 15.08 ± 7.27 years; controls: 14.04 ± 2.72 years). SCBT were shorter (SCBT: 151.13 ± 25.22 cm; controls: 162.19 ± 15.13) and had lower weight (SCBT: 53.32 ± 24.58 kg; controls: 60.09 ± 21.97) compared to non-cancer controls.

**Table 1:** Study Population Characteristics.

Variables	SCBT (n = 74)	Controls (n = 126)	P-value
	Mean ± SD	Mean ± SD	
Age at enrollment (years)	15.08 ± 7.27	14.04 ± 2.72	0.633
Sex, No. (%)			
Male	42 (56.80)	67 (53.20)	—
Female	32 (43.20)	59 (46.80)	—
Height (cm)	151.13 ± 25.22	162.19 ± 15.13	<0.001
Height z-score	−0.24 (−2.6–1.56)	0.28 (−2.01–1.99)	<0.001
Weight (kg)	53.32 ± 24.58	60.09 ± 21.97	0.003
Weight z-score	−0.10(−1.69–2.23)	0.20 (−1.45–4.42)	0.001
BMI percentile (%)	63.54 ± 30.83	63.46 ± 30.37	0.981
Fat mass percentage (%FM) (n = 180)	24.99 ± 9.99	22.68 ± 9.79	0.105
Waist-to-hip ratio (n = 198)	0.87 ± 0.07	0.83 ± 0.10	0.001
Waist-to-height ratio (n = 198)	0.48 ± 0.07	0.45 ± 0.08	0.014
Leptin (ng/ml; n = 47 SCBT, n = 97 controls)	14.74 ± 21.76	10.62 ± 12.11	0.770

Abbreviations: SCBT, survivors of childhood brain tumors; SD, standard deviation; BMI, Body Mass Index. The data for height and weight z-scores are reported a mean with range.

There were no differences in BMI percentiles in the two groups (SCBT: 63.54 ± 30.83; controls: 63.46 ± 30.37).

On assessment of adiposity phenotype in participants, total adiposity trended higher in survivors compared to non-cancer controls measured via fat mass percentage yet this was not statistically significant (%FM; SCBT: 24.99 ± 9.99%; controls: 22.68 ± 9.79%, p-value 0.105). In addition, SCBT had higher central adiposity compared to non-cancer controls including waist-to-hip ratio (WHR; SCBT: 0.87 ± 0.07, controls: 0.83 ± 0.10, p-value 0.001) and waist-to height ratio (WHtR; SCBT: 0.48 ± 0.07; controls: 0.45 ± 0.08, p-value 0.014). The majority of both groups have either completed or were undergoing pubertal development (SCBT n = 51, 68.9%, controls n = 109, 86.5%, p-value 0.005).

### Brain tumor characteristics

The characteristics of the tumors in SCBT group are presented in Table 2. The most common tumor type was low grade glioma (n = 42, 56.80%). Tumors were distributed equally between the supratentorial (n = 35, 47.30%) and infratentorial (n = 39, 52.70%) regions. The majority of survivors were surgically treated (n = 57, 77.00%), and some had radiotherapy (n = 30, 40.50%) and chemotherapy (n = 36, 48.60%) as per standard protocols^10,32,33^. Leptin levels correlated with surgery (r = 0.35, p-value 0.015) and radiotherapy (r = 0.43, p-value 0.002) but not chemotherapy (r = 0.18, p-value 0.230).

**Table 2:** Brain tumor characteristics (n = 74).

Variables	No. (%)
**Brain tumor type**	
Non-NF-1, low grade glioma	31 (41.90)
PNET/Medulloblastoma	16 (21.60)
NF-1, low grade glioma	11 (14.90)
CNS germ cell tumors	6 (8.10)
Subependymal giant cell astrocytoma	3 (4.10)
Ependymoma	2 (2.70)
Meningioma	1 (1.40)
Craniopharyngioma	2 (2.70)
Other	2 (2.70)
**Brain tumor location**	
Supratentorial	35 (47.30)
Infratentorial	39 (52.70)
**Brain tumor treatments**	
Surgery	57 (77.00)
Radiotherapy	30 (40.50)
Chemotherapy	36 (48.60)

Abbreviations: CNS, Central Nervous System; PNET, Primitive Neuroectodermal Tumor; NF-1, Neurofibromatosis Type 1.

### Leptin and adiposity measures in SCBT

To determine if leptin levels were different between SCBT and non-cancer controls, we measured plasma leptin levels using Enzyme Linked Immunosorbent Assay (ELISA) technique. The average leptin levels were similar between the two groups (SCBT: 14.74 ± 21.76 ng/ml vs controls: 10.62 ± 12.11 ng/ml, p = 0.770).

To determine if leptin was associated with the fat mass, we performed an unadjusted Pearson zero-order correlation analysis and an age, sex, and puberty-adjusted partial correlation analysis (Table 3). Leptin correlated with total adiposity (Unadjusted r = 0.68; Adjusted r = 0.67; p < 0.001). Leptin also correlated with central adiposity measures including a weak positive correlation with WHR (Unadjusted r = 0.19; Adjusted r = 0.29; p < 0.001) and a strongly positive correlation with WHtR (Unadjusted r = 0.58; Adjusted r = 0.63; p < 0.001).

**Table 3:** Regression analyses and correlations of predictors of leptin adjusted for age, sex, puberty, and cancer status.

Variable	Standardized coefficient β	Correlations		p-value
		Unadjusted Zero-order	Adjusted Partial	
**Dependent Variable: %FM**				
Leptin	0.67	0.68	0.67	<0.001
Cancer vs. Control Group	0.18	0.09	0.26	<0.001
**Dependent Variable: Waist-to-hip ratio**				
Leptin	0.30	0.19	0.29	<0.001
Cancer vs. Control Group	0.24	0.25	0.26	<0.001
**Dependent Variable: Waist-to-height ratio**				
Leptin	0.67	0.58	0.63	<0.001
Cancer vs. Control Group	0.17	0.17	0.22	<0.001

Abbreviations: BMI, Body Mass Index; CI, confidence interval; %FM, fat mass percentage.

To assess if leptin was associated with the fat mass in SCBT, we conducted multivariable linear regression analyses (Table 3). Leptin was associated with total adiposity (%FM β = 0.67, p < 0.001) and central adiposity (WHR β = 0.30, p < 0.001; WHtR β = 0.67, p < 0.001). Furthermore, having a brain tumor was associated with having a higher %FM (β = 0.18, p < 0.001) as well as central adiposity (WHR β = 0.24, p < 0.001; WHtR β = 0.17, p < 0.001). Treatments including surgery, radiotherapy, or chemotherapy had no effect on the association between adiposity and leptin levels (data not shown).

Taken together, these data demonstrated that leptin was a biomarker of total and central adiposity in SCBT and in non-cancer controls.

## Discussion

Up to 80% of children diagnosed with certain subtypes of brain tumors today are likely to survive their diagnosis^34^, yet the emergence of cardiometabolic disorders in survivors may undermine these survival rates and contribute to premature mortality^35–40^. Identifying biomarkers of the fat mass in SCBT may help predict who is at risk of excess adiposity, a known risk factor for the development of cardiometabolic disorders. The prediction of adiposity may allow risk stratification and the targeting of those in need of early aggressive interventions to improve survival and quality of life in survivors.

We demonstrate that leptin was a robust biomarker of total and central adiposity in SCBT, and that this trend was similar to the one noted in the non-cancer control group. To our knowledge, this is the first report of leptin assessment in SCBT in comparison to a non-cancer control group across a range of BMIs and adiposity levels.

In one group of brain tumors, Craniopharyngioma, it has been reported that patients develop hypothalamic obesity and hyperleptinemia^41,42^. The latter study by Shaikh *et al*. included obese participants with additional subtypes of brain tumors beside Craniopharyngioma, as well as a non-brain tumor group e.g. Histiocytosis, Retinoblastoma. In a cross-sectional design, the investigators used DXA scans to compare adiposity in the tumors group with two other groups-congenital hypopituitarism and simple obesity. The study had a smaller sample size when compared to our study^42^. The direct comparisons between our data and this study were limited due to these differences.

Leptin is a 16 kDa peptide hormone secreted mainly by the adipocyte and is encoded by the obese (OB) gene in humans that is located on chromosome 7^23,43^. The leptin receptor is preferentially expressed in hypothalamic nuclei including the ventromedial and dorsomedial nuclei, and the arcuate nucleus^44–46^, where it plays a critical role in regulating energy homeostasis through its role in satiety regulation and metabolic rate^23,47–50^. Excess caloric consumption raises leptin levels which increases energy expenditure while suppressing appetite^51,52^.

Leptin levels are also sensitive to changes in adiposity^53,54^. Weight loss leads to a reduction in leptin concentrations, likely due to a reduction in adipose tissue production of the adipokine^53,54^, and the opposite effect is seen in obesity^55^. Accordingly, leptin levels positively correlate with BMI, waist circumference and total adiposity in the general pediatric and adult populations^24–26,56,57^. Furthermore, females have higher circulating leptin levels compared to males in children and adults^58,59^. This makes leptin a potential biomarker of the response to interventions that target adiposity in SCBT.

While genetic leptin deficiency in humans is associated with early onset obesity^60,61^, leptin resistance at a hypothalamic level may play an important role in the development of diet-induced obesity^62–65^.

Leptin has also served as a biomarker for cardiometabolic outcomes^24,25,27–31,56^. Leptin levels positively correlate with fasting insulin concentrations^25^, and it is a predictor of glucose intolerance, insulin resistance and the metabolic syndrome independently of baseline obesity in the general population^29^. In men, increased leptin levels are a predictor for developing diabetes independently of basal adiposity, insulin resistance, glucose or age^27^. Also, elevated levels of leptin have been shown to be a significant predictor of coronary events^30^ and hypertension^31^.

In children, leptin is also a predictor of BMI, fasting insulin and triglycerides^57^.

Further research is required to determine if leptin can similarly be used as a potential biomarker to predict future cardiometabolic outcomes in the SCBT population similarly to the general population.

Leptin is secreted in proportion to the body’s fat mass, and reductions in its levels may induce over-feeding and weight gain^66–68^. However, the potential of leptin as a therapeutic weight-loss agent is limited since exogenous leptin delivery is associated with resistance to its effects and induces only mild physiological responses during diet-induced obesity^66–68^. In humans, obesity is not linked to leptin deficiency but rather to leptin insensitivity and factors that may improve leptin sensitivity have been studied^69^. For example, Amylin is a hormone that is co-secreted from beta cells along with insulin and is a powerful leptin stabilizer^70,71^. Additionally, the Glucagon-Like Peptide-1 Receptor (GLP-1R) agonist Exendin-4^72^, agonists of the melanocortin 4 receptor (MC4R)^73^ and the gut hormones PYY and Cholecystokinin^74,75^ have all shown success in eliciting sensitization of central leptin actions. Future pediatric studies should focus on understanding the interactions between leptin and these hormones in an attempt to decipher the mechanisms of leptin action and potential augmentation strategies that may be clinically relevant.

The inclusion of a non-cancer control group in comparison with SCBT, and the similar results noted between groups provides confidence in the results and indicate that leptin is a useful adiposity biomarker in SCBT.  The determination that leptin is a predictor of total and central adiposity in SCBT is novel and provides a baseline for future studies of adiposity in this population.

One of the limitations of this study is the lack of long-term follow-up data regarding the association of leptin with long-term cardiometabolic outcomes. Longitudinal follow-up and a sample size that allows for subgroup analyses based on tumor subtype will help predict which groups are at risk of adverse cardiometabolic outcomes to allow early intervention.

In conclusion, this cross-sectional study demonstrated that leptin is a biomarker of total and central adiposity in SCBT. Further investigation into leptin as a potential marker of future cardiovascular disease and type 2 diabetes in SCBT is needed.

 It may also help stratify those in need of early interventions to prevent and treat cardiometabolic disorders in this population of cancer survivors.

## Methods

### Participants

The complete study methodology has been reported previously^76,77^. This is a secondary analysis of cross-sectional data from the Canadian Study of Determinants of Endometabolic Health in Children (CanDECIDE study)^76,77^. The Hamilton Integrated Research Ethics Board has approved this project. Study procedures were carried out in accordance with the relevant guidelines and legal regulations. Male and female participants, who were 5 years or older, were consecutively recruited from McMaster Children’s Hospital (Hamilton, Ontario, Canada) from November 2012 to December 2016. participants of all ethnicities with no history of autoimmune diseases or infections or having been treated with immunosuppressive therapy within 15 days of participation were eligible for recruitment into the study.

All participants provided written informed consent. Participants 16 years and older provided their own consent. For participants between 7–15 years of age, assent as well as parental/guardian consent was obtained. Participants under 7 years of age were included in the study with parental consent.

### Anthropometric and clinical measurements

Data on age, sex, puberty, and ethnicity were collected using standardized questionnaires. To determine the medical history of SCBT, including diagnostic and treatment data, we consulted medical records.

Height was measured to the closest 0.1 cm using a stadiometer and weight to the nearest 0.1 kg with an electronic scale (Seca, USA). Weight and height measurements were used to determine BMI (kg/m^2^). Furthermore, BMI percentile and BMI z-score were classified using the Children’s BMI Tool for Schools^78^ and the Centers for Disease Control and Prevention (CDC) growth chart^79^, respectively. The Tanita body fat monitor (Tanita Corporation, Illinois, USA) was used to measure fat mass percentage (%FM) to determine adiposity in participants^15^.

### Quantification of plasma leptin levels using ELISA

Fasting plasma samples were collected by centrifuging EDTA-treated whole blood at 1,500 g for 15 minutes at room temperature. Plasma samples were aliquoted and stored in cryovials at −80 °C until further use. On the day of the assay, plasma samples were thawed on ice and centrifuged once at 1,500 g for 15 minutes at room temperature. Plasma samples were diluted, and leptin levels were quantified using the commercially available enzyme linked immunosorbent assay (ELISA), Human Leptin Quantikine ELISA Kit (R&D Systems, Minneapolis, USA) as per manufacturer’s guidelines.

### Statistical analysis

SPSS versions 24.0 and 25.0 were used to perform all statistical analyses^80^. Categorical variables are presented as counts (%) and continuous variables are reported as means (SD). Outliers were determined using visual inspection and box plots, and the Shapiro-Wilk test was used to determine normality of the data distribution^81^. Non-normally distributed data were log-transformed for inclusion in the analyses. In the case of missing data, multiple imputations were used.

Correlation analyses were conducted using a Pearson zero-order correlation test and partial correlation test unadjusted and age, sex, and puberty-adjusted values. Multivariable linear regression analysis was used to determine whether leptin is associated with total or central adiposity with the independent variables including age, sex, puberty, and cancer status. Results are reported as standardized *β* coefficients and associated p-values. We also conducted unstandardized coefficients testing with resulting B values with 95% confidence intervals (CI) and p-values. Since the results for both trended in the same direction, we report only the results of the standardized testing in Table 3. Statistical significance was set at alpha = 0.05.
